# Genome-wide analysis of cancer cell-derived Foxp3 target genes in human tongue squamous cell carcinoma cells

**DOI:** 10.3892/ijo.2015.2926

**Published:** 2015-03-13

**Authors:** YU-JIE LIANG, XIAO-MEI LAO, LI-ZHONG LIANG, GUI-QING LIAO

**Affiliations:** 1 Department of Oral and Maxillofacial Surgery, Guanghua School of Stomatology, Guangdong Provincial Key Laboratory of Stomatology, Sun Yat-sen University, Guangzhou, P.R. China; 2 Department of Oral and Maxillofacial Surgery, Fifth Affiliated Hospital of Sun Yat-sen University, Zhuhai, P.R. China

**Keywords:** Foxp3, tongue squamous cell carcinoma, regulatory T cells, chromatin immunoprecipitation with DNA microarray, genome

## Abstract

The forkhead transcription factor Foxp3 is essential for differentiation and activation of regulatory T cells (Tregs), and used to be regarded as specific transcription factor of Tregs. In recent years, Foxp3 expression in tumor cells (cancer cell-derived Foxp3) has gained great interest, but its function and molecular mechanisms remain incompletely understood. In the present study, we detected dynamic nuclear translocation of Foxp3 in TSCC cells using immunofluorescent staining. Then we performed a genome-wide analysis of Foxp3 in TSCC cells using a combination of ChIP-on-chip and whole-genome microarray assays. We also compared Foxp3 biding sites in TSCC cells with the known binding sites in human Tregs to show the differences in transcriptional regulation profile. Results indicate that Foxp3 in TSCC cells has distinct biological functions compared with that in Tregs. Cancer cell-derived Foxp3 directly regulates the transcription of genes that affect certain internal biological processes of TSCC cells, and indirectly influences the extracellular microenvironment. This study reveals the relationship between direct and indirect targets genes of Foxp3 in TSCC cells and provide molecular basis of cancer cell-derived Foxp3 function.

## Introduction

Regulatory T cells (Tregs) are key players in maintaining immune homeostasis and tolerance. The forkhead transcription factor Foxp3 is essential for differentiation and activation of Tregs (1), and used to be regarded as specific transcription factor of Tregs (2,3). In 2007, Hinz *et al* (4) first reported that pancreatic cancer cells expressed Foxp3. Subsequent studies reported that breast cancer cells expressed Foxp3, and that Foxp3 positivity was associated with poor prognosis (5). However, other studies reported that Foxp3 acts as a tumor suppressor in breast cancer and prostate cancer (6–8). Thus, the role of Foxp3 expression in cancer cells (referred as ‘cancer cell-derived Foxp3’ in this report) remains incompletely understood, especially regarding molecular mechanisms.

At the molecular level, FOXP3 binds to multiple transcription factors, such as NFAT, NF-κB, STAT3, AML1/Runx1 to regulate T cells function (9–12). It also modulates gene expression through epigenetic mechanisms, such as chromatin remodeling and histone deacetylation (13,14). Zheng *et al* (15) first performed a genome-wide analysis of Foxp3 in mouse Tregs and found that Foxp3 acts as both a transcriptional activator and repressor in Tregs. Recently, Rudra *et al* (16) reported that Foxp3 binds to 361 proteins in Tregs and is involved in the transcriptional regulation of most of these proteins. The above demonstrate a complex nature of the interaction of Foxp3 with its target genes. However, less is known about the role of Foxp3 in the transcriptional regulation in cancer cells. In particular, it is unknown whether Foxp3 regulates transcription in cancer cells as it does in Tregs.

Our previous study revealed the expression of Foxp3 in tongue squamous cell carcinoma (TSCC) cells, and showed that the expression of cancer cell-derived Foxp3 was positively associated with the pathologic differentiation and T stage, and inversely associated with overall survival of TSCC patients (17). To achieve further knowledge on these influences, and how cancer cell-derived Foxp3 can regulate TSCC, the present study was performed, using genome-wide analysis of Foxp3 target genes in TSCC cells with a combination of chromatin immunoprecipitation array profiling (ChIP-on-chip assay) and expression profiling (whole-genome microarray assay). We also compared Foxp3 biding sites in TSCC cells with the known binding sites in human Tregs to show the differences in transcriptional regulation profile. This study revealed the relationship between direct and indirect targets genes of Foxp3 in TSCC cells and provide molecular basis of cancer cell-derived Foxp3 function.

## Materials and methods

### Cell cultures

Three human TSCC cell lines (CAL 27, SCC-9, and SCC-5) were purchased from American Type Culture Collection (ATCC). CAL 27 cells were maintained in DMEM (Gibco, Grand Island, NY, USA) that contained 10% fetal bovine serum (FBS) (Gibco). SCC-9 cells and SCC-5 cells were maintained in DMEM/F-12 (Gibco) that contained 10% FBS.

### Cytoimmunofluorescence staining

CAL 27, SCC-9, and SCC-5 cells were seeded into 48-well plates for routine culturing. After washing in PBS, cells were fixed in 4% formaldehyde for 20 min at room temperature, treated with 1% Triton, and then blocked in 5% bovine serum albumin (BSA) at room temperature for 50 min. The cells were then incubated with goat anti-human Foxp3 antibody (10 μg/ml, R&D Systems, Minneapolis, MN, USA) at 4°C overnight and Northern Lights anti-goat IgG-NL557 (1:200, R&D Systems) at room temperature in the dark for 1 h. After nuclear staining with 5 μg/ml DAPI for 1 min, cells were observed under an inverted microscope (Axio observer Z1, Zeiss). Negative control was performed by replacing the primary antibody with PBS.

### ChIP-on-chip and bioinformatics analysis

SCC-9 cells were seeded into 6-well plates and cultured for 48 h. After washing in PBS twice, 2 ml of fresh medium and 54 μl of 37% formaldehyde were added to each well, followed by incubation at room temperature for 10 min. Then, 200 μl of glycine was added, followed by incubation for 5 min at room temperature. The medium was removed and cells were washed twice with pre-chilled 5 mM EDTA. Then, 200 ml of PBS with 1% PMSF was added to each well, and the cells were harvested. The ChIP-on-chip assay (Shanghai Kangcheng Biotech Co., Ltd., Shanghai, China) was performed with goat polyclonal antibody against FOXP3-ChIP Grade (Abcam, Hong Kong, China) and NimbleGen HG18 3×720K RefSeq promoter microarray (Roche, Mannheim, Germany). Gene ontology (GO) and pathway analysis were performed with the cooperation with Shanghai Kangcheng Biotech Co., Ltd. The binding sites of FOXP3 in the genome of human Tregs were obtained from the ChIP-on-chip results of Sadlon *et al* (18).

### Gene silencing with siRNA

SCC-9 cells were routinely cultured. When the confluence reached 30–50% (24 h), cells were harvested and treated with 50 nM siRNA (Guangzhou RiboBio Co., Ltd., Guangzhou, China) and an equal amount of Lipofectamine 2000 (Invitrogen, Carlsbad, CA, USA), followed by incubation for 5 h. At 24 and 48 h after transfection, total RNA and protein were extracted for real-time PCR and western blot assays to assess the effect of Foxp3 on interference. The siRNA sequences were described earlier (4,17).

### Western blot assays

As described in our earlier study (17), cells were harvested in RIPA buffer (Beyotime, Shanghai, China), and protein concentration was determined by Bradford assay (Bio-Rad Laboratories, Shanghai, China). Equal amounts of total protein were subjected to 10% SDS-PAGE and then transferred onto PVDF membranes (Millipore, Billerica, MA, USA). The membrane was blocked in Tris-buffered saline (TBST) containing 5% BSA for 2 h at room temperature, and then incubated with 1.0 μg/ml anti-human Foxp3 antibody (R&D Systems) at 4°C overnight. After washing in TBST, the membrane was incubated in horseradish peroxidase-conjugated anti-goat IgG (1:10,000; Santa Cruz Biotechnology, Inc. (Santa Cruz, CA, USA) for 1 h. Each sample was probed with an anti-GAPDH antibody (1:1,000; Santa Cruz) as a loading control.

### RNA extraction, reverse transcription and real-time PCR

Total RNA was extracted with the High Pure RNA Isolation kit (Roche) according to the manufacturer’s instructions. RNA (1 μg of each sample) was then used for reverse transcription into cDNA with the Transcriptor First Strand cDNA Synthesis kit (Roche) according to the manufacturer’s instructions. The mRNA expression of Foxp3 was examined by real-time PCR using Light-Cycler 480 SYBR Green I Master (Roche, Mannheim, Germany) and the thermal cycling conditions and primers were the same as described in our earlier study or selected from PrimerBank (17,19). PCRs were conducted in triplicate for each sample. GAPDH was used as internal reference and the 2^−ΔΔCt^ method was used to determine gene expression.

### Human genome-wide expression profiling

After silencing with siRNA for 48 h, RNA in SCC-9 cells was extracted, and the Human Genome U133 plus 2.0 array (Affymetrix, USA) was used for the whole genome array assay. Microarray hybridization was carried out at CapitalBio Corp. (Beijing, China). Cluster analysis was performed with Cluster 3.0 software. Data analysis was performed using Significance Analysis of Microarray software (SAM 3.0, Stanford University, USA; http://www-stat.stanford.edu).

### Statistical analysis

Statistical analysis was carried out using SPSS 17.0 statistical software package. Quantitative data analysis employed Student’s t-test to compare two groups and one-way analysis of variance (ANOVA) to compare multiple groups. A P-value <0.05 was considered statistically significant.

## Results

### Translocation of Foxp3 into nuclei of TSCC cells

**Figure 1:**
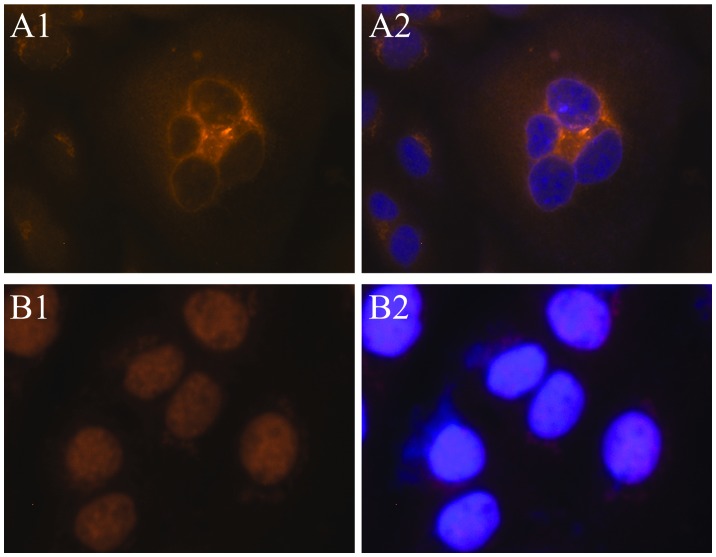
Subcellular translocation of Foxp3 in SCC-9 cells detected by immunofluorescence staining (×400). (A1 and B1) Foxp3 staining; (A2 and B2) Foxp3 staining merged with DAPI staining. When SCC-9 cells were cultured for 24 h, Foxp3 distributed in the whole cell parts, with the greatest concentration at the nuclear membrane, which appeared as a round fluorescent body (A). When SCC-9 were cultured for 48 h, Foxp3 concentrated in nucleus, and the round fluorescent body disappeared, which showed a dynamic nuclear translocation (B).

**Figure 2:**
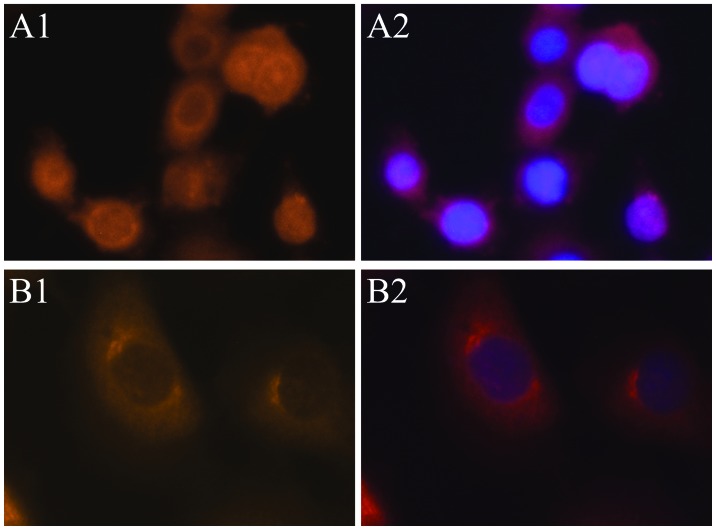
Subcellular distribution of Foxp3 in CAL27 and SCC-15 cells detected by immunofluorescence staining (×400). (A1 and B1) Foxp3 staining; (A2 and B2) Foxp3 staining merged with DAPI staining. When CAL27 cells (A) and SCC-15 cells (B) were cultured for 24 h, Foxp3 expressed in the whole cell parts, with the greatest concentration at the nuclear membrane.

Nuclear translocation is essential for transcription factor function, so at first we used immunofluorescent staining to make clear the subcellular distribution of Foxp3 in TSCC cells. After culture of SCC-9 cells for 24 h, the immunofluorescence showed that Foxp3 was present throughout the cells, with the greatest concentration at the nuclear membrane, which appears as a round fluorescent body (Fig. 1A). This expression pattern could also be observed in SCC-15 and CAL 27 cells (Fig. 2). After 48 h, Foxp3 was mainly expressed within the nucleus, and the round body shape of fluorescent staining at the nuclear membrane disappeared (Fig. 1B), indicating that Foxp3 was transported into nucleus gradually.

**Figure 3:**
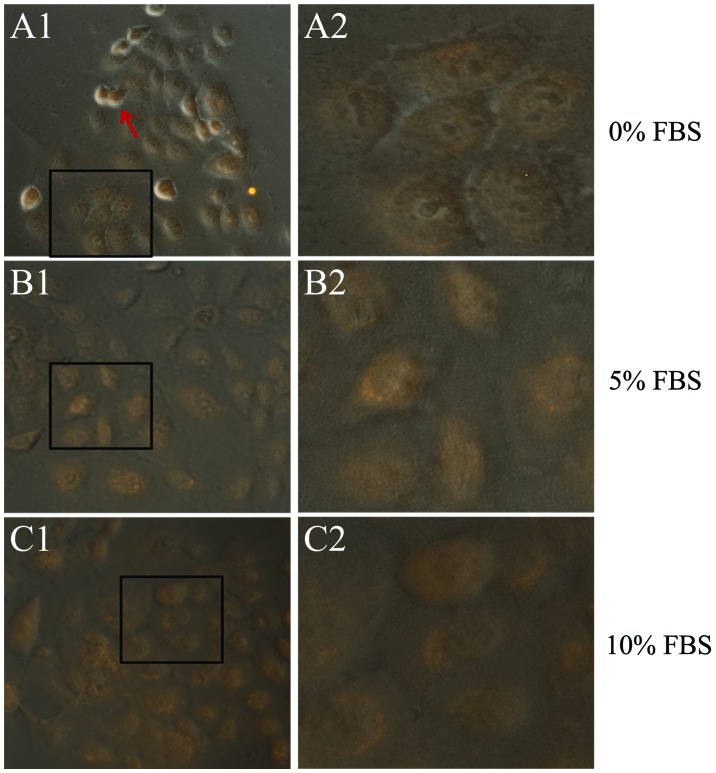
SCC-9 cells cultured for 24 h in DMEM-F12 medium with 0, 5 and 10% FBS, respectively. Phase contrast images merged with Foxp3 immunofluorescence staining. Cells in 0% FBS displayed a worse growth, and some cells became round and suspended (A1, red arrows). Cells in 5 and 10% FBS displayed similar normal growth (B and C). Cells in each group had Foxp3 nuclear distribution (A–C).

Next, SCC-9 cells were cultured in DMEM-F12 that contained 0, 5 and 10% FBS for 24 h and then underwent Foxp3 immunofluorescent staining. It can be observed that cells in the 0% FBS group had a poor growth, and some cells became round and suspended. However, there was Foxp3 expression in the nuclei of cells in all three groups (Fig. 3). This culture assay shows that stimulation from other cell types are not needed in the nucleus translocation of Foxp3 in TSCC cells.

### Foxp3 binding sites and functional annotation in the genome of TSCC cells

The ChIP-on-chip assay identified 4140 binding sites of Foxp3 in the genome of SCC-9 cells [false discovery rate (FDR) <0.05]; after accounting for identical genes, there were 3573 Foxp3-binding genes. Among all genes with FDR values <0.005, there were 25 transcriptional factors: POU3F1, HEY1, TEAD1, POU4F2, VEZF1, KCNIP4, KLF12, E2F1, REST, FOXO4, NR4A1, HOXB8, POU2F1, HOXD9, HIC1, ZBTB16, TCF7, KLF11, IGFBP7, NFIC, PKNOX1, TWIST1, ING1, MEF2A and LITAF.

**Figure 4:**
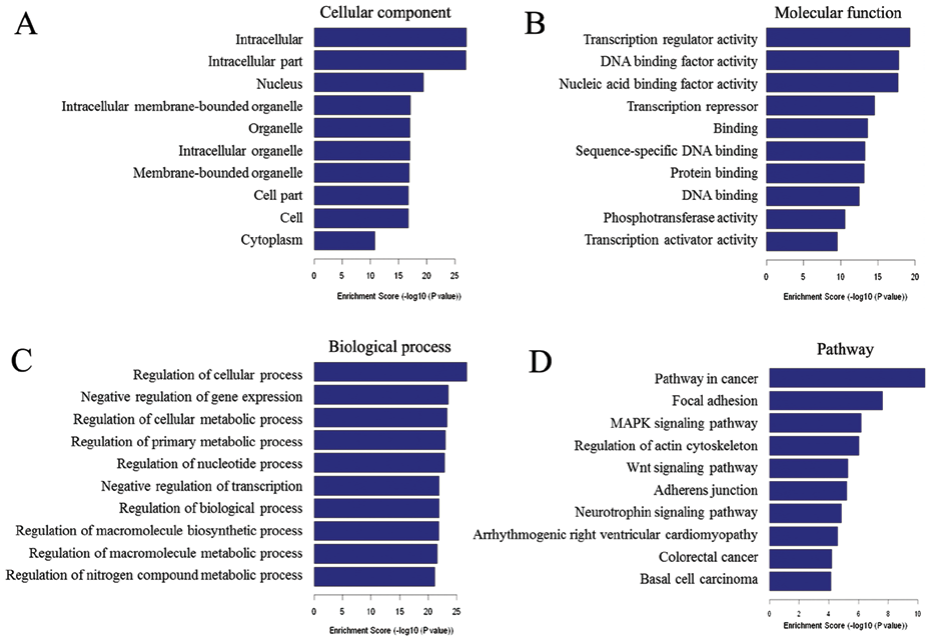
GO analyses of Foxp3-biding genes in human TSCC cell genome. The top 10 significant GO terms and their enrichment scores was displayed. Cell component analysis showed that the proteins encoded by Foxp3-binding genes mainly located in intracellular parts of TSCC cells (A). Molecular function analysis showed that the proteins encoded by Foxp3-binding genes mainly functioned in transcriptional regulation and biological macromolecule biding (B). Biological analysis showed that the proteins encoded by Foxp3-binding genes were involved in many general regulations (C). Pathway analysis in KEGG database indicated that 9 of the top 10 pathways were associated with cancer (D).

**Figure 5:**
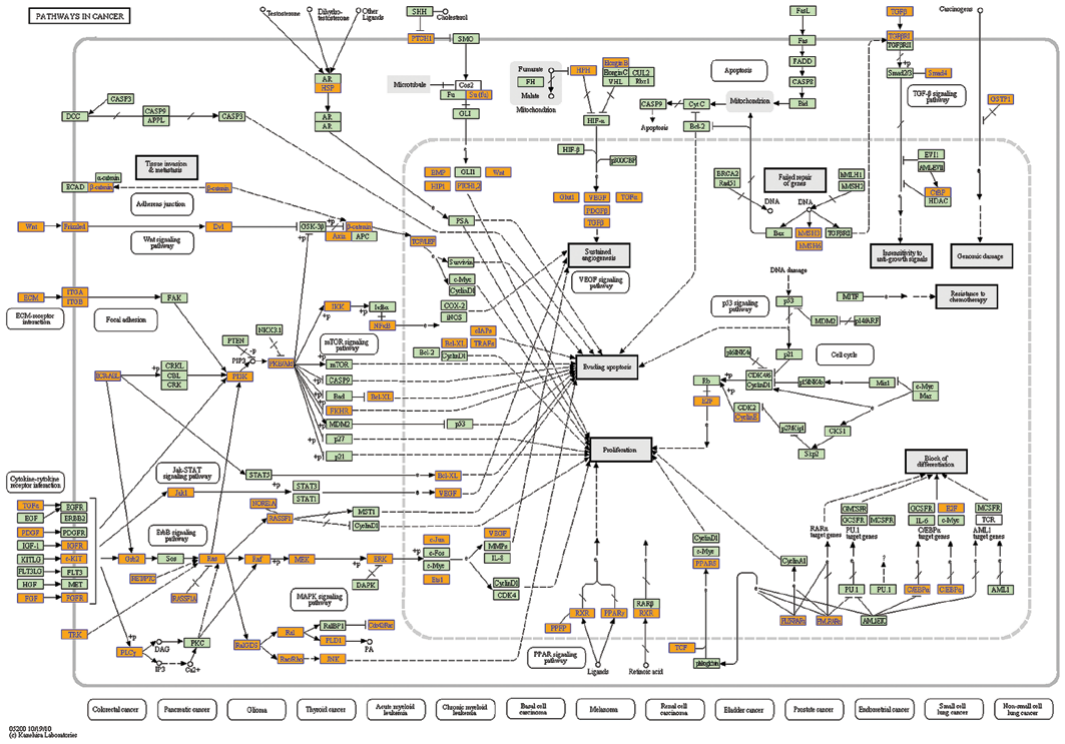
Foxp3-biding genes in ‘pathway in cancer’. Pathway in cancer is the top 1 pathway in pathway analysis of Foxp3-binding genes in TSCC cells. Positions in red are Foxp3-biding genes. Cancer cell-derived Foxp3 strongly participate in the regulation network of cancer.

To reveal general functional features of the molecular program implemented by Foxp3 in TSCC cell, we conducted GO analysis of Foxp3-binding genes. Results showed that the proteins encoded by Foxp3-binding genes mainly located in intracellular parts of TSCC cells (top 10 GO terms - cellular component, Fig. 4A), functioned in transcriptional regulation and biological macromolecules biding (top 10 GO terms - molecular function, Fig. 4B). Biological analysis showed that the proteins encoded by Foxp3-binding genes were involved in many general regulations (top 10 GO terms - biological process, Fig. 4C), and in the top 324 GO terms with P-values <0.001, 131 terms (40.43%) were associated with transcriptional regulation, both upregulation and downregulation. Analysis of our data with the KEGG database indicated that 9 of the top 10 pathways were associated with cancer (Figs. 4D and 5).

### Comparison of Foxp3-binding genes in human TSCC cells and Tregs

**Figure 6:**
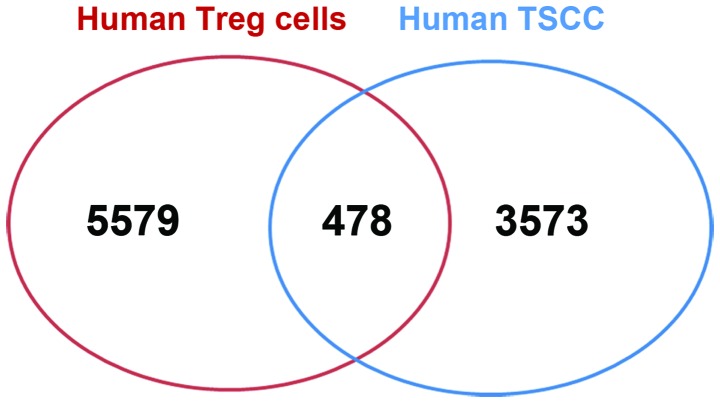
Overlap between Foxp3-binding genes in human TSCC cells and Tregs. The numbers present binding genes common (center) and unique to each cell type (outer). This overlap corresponds to 12.28% of the Foxp3-binding genes in human TSCC cells and 8.75% of the Foxp3-binding genes in Tregs.

We compared the Foxp3-binding genes in human TSCC cells with the known Foxp3 binding sites in human Tregs (18). Previous ChIP-on-chip data showed that 5,579 genes were bound by Foxp3 in Tregs (18). Comparison results showed that 478 Foxp3-binding genes in our ChIP-on-chip data set were also Foxp3-binding genes in human Tregs. This overlap corresponds to 12.28% of the Foxp3-binding genes in human TSCC cells and 8.75% of the Foxp3-binding genes in Tregs (Fig. 6).

GO analysis of these 478 overlapped genes showed similar results with the Foxp3-binding genes in human TSCC cells. The encoded proteins were mainly localized on the cell membrane and in the intracellular parts, and functioned in the regulation of transcription and binding to nucleic acids or proteins, including NF-κB (P=4.22×10^−8^), but rarely involved in T cell-specific biological processes.

Previous analysis indicated that Foxp3-binding genes in Tregs take part in 86 pathways, most of which are associated with the differentiation, activation, and death of T cells under normal and pathological conditions. However, pathway analysis of overlapped genes also showed similar results with the Foxp3-binding genes in human TSCC cells, and 7 of the top 10 pathways were involved in cancer-related pathways.

### Effect of Foxp3 on gene expression in human TSCC cells

**Figure 7:**
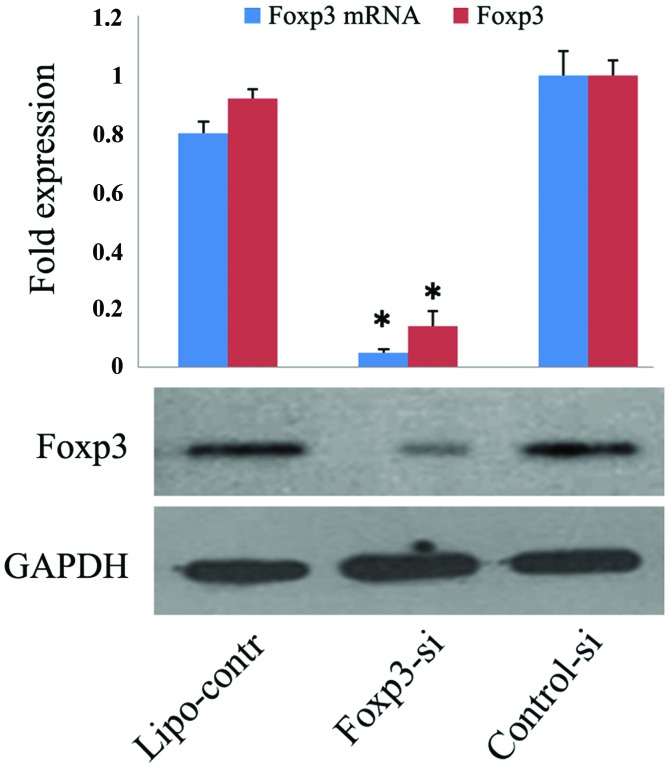
Downregulation of Foxp3 expression in SCC-9 cells using siRNA. Real-time PCR showed Foxp3 mRNA decreased 95%. Western blotting showed Foxp3 protein expression decreased 85.8%.

Next, we used siRNA to downregulate Foxp3 expression in TSCC cells. In these experiments, SCC-9 cells were transfected with Foxp3 siRNA (Foxp3-si group), control-siRNA (control-si group), or Lipofectamine 2000 (lipo-control group). At 48 h after transfection of SCC-9 cells, Foxp3 expression was downregulated by 85% in the Foxp3-si group relative to the control-si group (P=0.001). There was no marked difference between control-si and lipo-control groups (Fig. 7).

**Figure 8:**
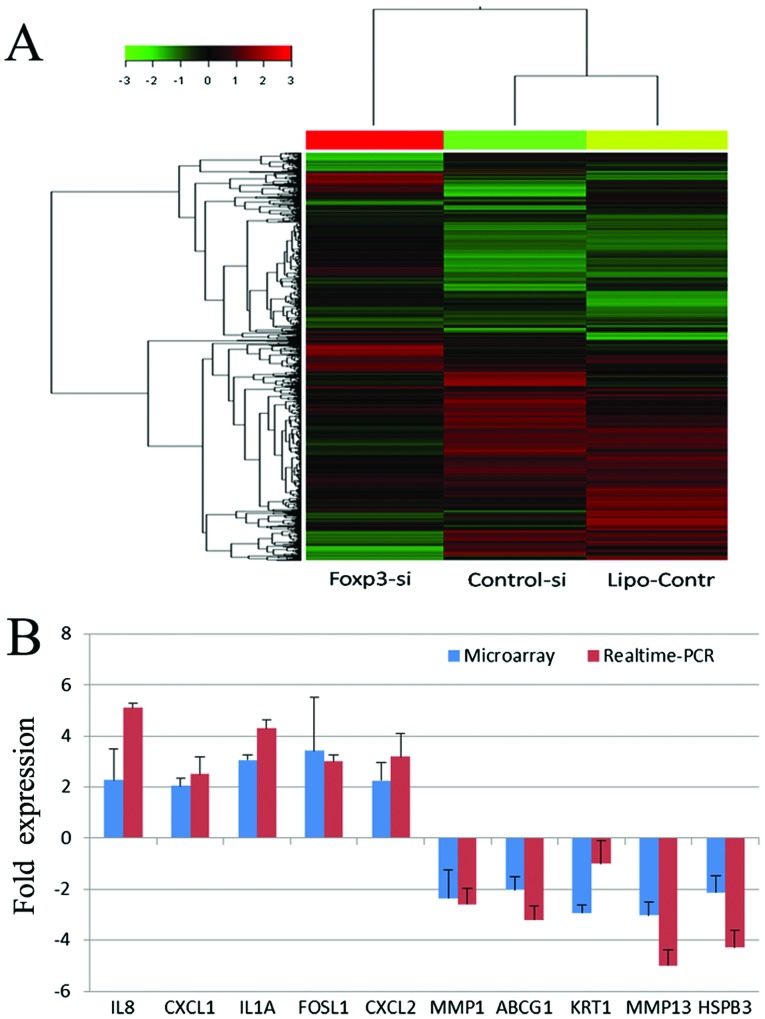
Human genome expression arrays of TSCC cells after Foxp3 RNAi. Cluster analysis showed that comparing to control-si group and lipo-control group, many genes were differently expressed in Foxp3-si group (A). Real-time PCR of 10 randomly selected differently expressed genes validated the microarray results (B).

Then human whole-genome microarray assay showed that there was no significant difference in the gene expression profiles of the control-si group and the lipo-control group, and that the Foxp3-si group was significantly different from the other two groups (Fig. 8A). When the cut-off ratio was set at 2-fold change in expression, 30 genes were upregulated and 36 genes were downregulated in the Foxp3-si group. When the cut-off ratio was set at 1.5-fold change in expression, 269 genes were upregulated and 330 genes were downregulated in the Foxp3-si group. Real-time PCR of 10 randomly selected different-expressed genes was performed to validate the microarray results, as shown in Fig. 8B.

**Figure 9:**
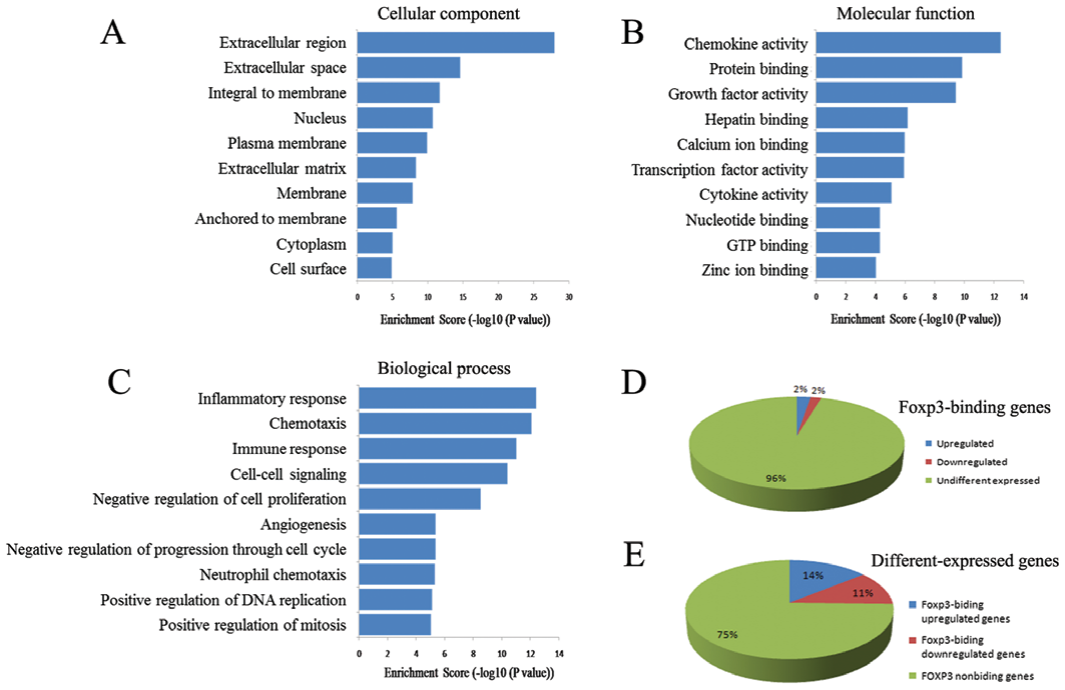
Analyses of differently expressed (DE) genes in human TSCC cell genome after Foxp3 RNAi. (A–C) The top 10 significant GO terms and their enrichment scores. Cell component analysis showed that the proteins encoded by DE genes were mainly distributed in the extracellular parts and the cell membrane (A). Molecular function analysis showed that besides some biological macromolecules biding functions, the proteins encoded by DE genes were closely related to the cytokine network in that they influenced chemokine activity, growth factor activity and cytokine activity (B). Biological analysis showed that the proteins encoded by DE genes were mainly associated with the regulation of the microenvironment and immunity, such as inflammatory responses, chemotaxis, immune responses, cell-cell signaling, angiogenesis and neutrophil chemotaxis (C). Cross-referencing the data set of Foxp3-binding genes and DE genes showed that 152 genes (associated genes) were identical in the ChIP-on-chip and expression profiling, with 85 genes being upregulated and 67 genes being downregulated. These associated genes accounted for 4.25% (152/3573) of the Foxp3-binding genes (D) and 25.38% (152/599) of the differently expressed genes (E).

We further performed bioinformatics analysis on these differently expressed genes. Cellular component analysis showed that the proteins encoded by differently expressed genes were mainly distributed in the extracellular parts and the cell membrane (Fig. 9A). Molecular function analysis showed that they were closely related to the cytokine network in that they influenced chemokine activity, growth factor activity, and cytokine activity (Fig. 9B). Analysis of the biological processes showed that the top 10 terms were mainly associated with the regulation of the microenvironment and immunity, such as inflammatory responses, chemotaxis, immune responses, cell-cell signaling, angiogenesis, and neutrophil chemotaxis (Fig. 9C).

Pathway analysis also showed that the proteins encoded by differently expressed genes mainly took part in pathways associated with cytokines and inflammatory reactions, such as cytokine-cytokine receptor interactions, adhesion and diapedesis of lymphocytes, adhesion and diapedesis of granulocytes, molecules involved in local acute inflammatory responses, cytokines and cytokine networks, and inflammatory responses.

### Direct regulation of gene transcription by Foxp3 in TSCC cells

The ChIP-on-chip and human genome-wide expression profiling assay showed significant difference between the differently expressed genes (after downregulation of Foxp3 expression) and Foxp3-binding genes in TSCC cells. Thus, we tried to reveal the correlation between the data set of ChIP-on-chip and profiling assay to identify genes that are directly regulated by Foxp3. After cross-referencing the data set of Foxp3-binding genes and differently expressed genes, results show that 152 genes (associated genes) were identical in the ChIP-on-chip and expression profiling, with 85 genes being upregulated and 67 genes being downregulated. These associated genes accounted for 4.25% (152/3573) of the Foxp3-binding genes (Fig. 9D) and 25.38% (152/599) of the differently expressed genes (Fig. 9E).

When these associated genes were further analyzed, results showed that the top GO term in cell component was the nucleus. Molecular functions focused on nucleic acid and protein biding, and had little association with the regulation of cytokines. Analysis of biological processes showed that these genes were similar to Foxp3-binding genes in TSCC cells, and that they are extensively involved in different biological processes. Notably, these genes were not specific for the regulation of cytokines, immune responses, inflammatory reactions, and the cellular microenvironment.

Pathway analysis by use of the KEGG database showed that pathways with P-values <0.001 included adherens junction (P=4.88×10^−4^), ECM-receptor interaction (P=5.83×10^−4^), small cell lung cancer (P=6.25×10^−4^), focal adhesion (P=6.77×10^−4^), and nitrogen metabolism (P=9.80×10^−4^). These results are also similar to those of FOXP3-binding genes in TSCC cells.

## Discussion

In the present study, we performed a genome-wide analysis of Foxp3 target genes in TSCC cells using a combination of ChIP-on-chip and whole-genome microarray assays. We identified direct and indirect target genes of cancer cell-derived Foxp3 for the first time. Our data suggest that cancer cell-derived Foxp3 directly regulates the transcription of genes that affect certain internal biological processes of TSCC cells, and indirectly influences the extracellular inflammatory micro-environment.

Foxp3 in Tregs is a well-known inducible transcriptional factor. In Tregs, Foxp3 mainly localize in the cytoplasm or adjacent to the nucleus when cells are unstimulated. Upon stimulation with the anti-CD3 or anti-CD28 antibody, Foxp3 undergoes T cell receptor (TCR)-mediated post-transcriptional modification, and within 1 h is translocated into the nucleus (13). However, additional factors may also be needed in Foxp3 translocation after TCR stimulation (20). Moreover, TCR is a specific receptor on the surface of T cells rather than other cell types. Therefore, it is necessary and still difficult to elucidate the mechanism of Foxp3 translocation in tumor cells. Our results showed that Foxp3 expressed in TSCC cells can enter the nucleus, even in the absence of serum. This suggests that cancer cell-derived Foxp3 entry into the nucleus is independent of exogenous stimuli. We speculate that non-microenvironment dependent signal peptide may exist in cancer cell-derived Foxp3, and that factors expressed by TSCC cells themselves may promote Foxp3 translocation into the nucleus, even that TSCC cells can secret factors into microenvironment to induce Foxp3 translocation in Tregs, which can be an exquisite ‘cross-talk’ between tumor cells and lymphocytes. Elucidation of the specific mechanism requires further investigation.

The capability of nucleus translocation of Foxp3 in these cell lines created basic conditions for genome study. We initially speculated that cancer cell-derived Foxp3 may directly regulate the transcription of some extracellular factors, such as cytokines and chemokines, similarly to Foxp3 in Tregs. However, when we used the ChIP-on-chip assay to identify Foxp3 binding sites in the genome of TSCC cells, bioinformatic analysis indicated that proteins encoded by these genes are mainly localized within TSCC cells, and many of these genes are involved in cancer related biological processes. In particular, analysis of molecular function showed that these proteins may bind to multiple proteins, including other transcriptional factors, and this may lead to co-regulation of related genes or alter the levels of free transcriptional factors and thereby affect transcription. Therefore, cancer cell-derived Foxp3 appears to regulate gene transcription through multiple patterns, such as direct regulation, regulation of other transcriptional factors, and regulation of proteins that bind to other transcriptional factors. In the study of Tregs, Rudra *et al* (16) also showed that, Foxp3 directly binds to genes and regulates the expression of proteins that bind to and regulate Foxp3 itself. This is the first report presenting the DNA binding profile of cancer cell-derived Foxp3. Pathway analysis further showed that the proteins encoded by Foxp3-binding genes are associated with cancer-related pathways.

Sadlon *et al* (18) performed ChIP-on-chip studies of Foxp3 in human Tregs in 2010 and the genes they identified were distributed in 86 pathways, most of which were associated with the functions and life activities of T cells. In this study, 11 of the pathways that we identified in TSCC cells were also present in Treg cells; four pathways with high enrichment were closely related to cancer. We also compared the Foxp3-binding genes in TSCC cells with those in Treg cells. The results showed that only 478 genes (13.38% of Foxp3-binding genes in TSCC cells) were in both TSCC cells and Tregs. These findings suggest that there are significant differences in the genes regulated by Foxp3 in Tregs and TSCC cells, and that cancer cell-derived Foxp3 has distinct biological functions.

We performed genome-wide expression profiling in TSCC cells after downregulation of Foxp3 by RNAi. The results confirmed that cancer cell-derived Foxp3 affected gene expression in TSCC cells. Analysis showed that the proteins encoded by these differently expressed genes were mainly distributed in the extracellular domain and cell membrane, and functioned to influence the extracellular microenvironment and inflammation, such as components of the extracellular matrix, intercellular signal transduction, activities of chemokines, growth factors and cytokines (including IL-8 signaling, down-regulation of IFN-γ and upregulation of IL-6). As cytokine network in tumor microenvironment affect Tregs proliferation and function (21,22), this part of findings are consistent with the hypothesis that cancer cell-derived Foxp3 regulates the microenvironment through its association with Tregs, thereby influencing the tumor immune response. Still, this hypothesis requires confirmation by further studies.

Notably, the proteins encoded by Foxp3-binding genes were mainly localized within TSCC cells. Therefore, the differently expressed genes and Foxp3-binding genes were significantly different in profile (only 25.38% overlap), protein distribution and function, which indicated that genes eventually influenced by cancer cell-derived Foxp3 are significantly different from those directly regulated by cancer cell-derived Foxp3. This suggests that cancer cell-derived Foxp3 indirectly affect the inflammatory microenvironment of TSCC.

However, the 25.38% overlap (associated genes) indicated that cancer cell-derived Foxp3 could also directly regulate gene transcription in TSCC cells. The results of bioinformatic analysis associated genes are similar to those of Foxp3-binding genes in TSCC cells and are widely involved in a variety of cellular processes, rather than the regulation of microenvironment and inflammation. These findings indicate that cancer cell-derived Foxp3 may directly regulate gene transcription and influence a fraction of biological processes in TSCC cells, and indirectly regulate gene transcription to affect the extra-cellular inflammatory microenvironment. Further studies such as dual-luciferase reporter gene assay and microenvironment co-cultured model could be used to confirm the mechanism of regulating specific genes by cancer cell-derived Foxp3.

In conclusion, we have, for the first time, identified direct and indirect target genes of cancer cell-derived Foxp3 in TSCC cells. Cancer cell-derived Foxp3 directly regulate the transcription of genes that affect certain internal biological processes of TSCC cells, and indirectly influence the extracellular microenvironment.

## References

[1] Marson A, Kretschmer K, Frampton GM, Jacobsen ES, Polansky JK, MacIsaac KD, Levine SS, Fraenkel E, von Boehmer H, Young RA (2007). Foxp3 occupancy and regulation of key target genes during T-cell stimulation. Nature.

[2] Gavin MA, Rasmussen JP, Fontenot JD, Vasta V, Manganiello VC, Beavo JA, Rudensky AY (2007). Foxp3-dependent programme of regulatory T-cell differentiation. Nature.

[3] Sakaguchi S (2010). Immunology: Conditional stability of T cells. Nature.

[4] Hinz S, Pagerols-Raluy L, Oberg HH, Ammerpohl O, Grüssel S, Sipos B, Grützmann R, Pilarsky C, Ungefroren H, Saeger HD (2007). Foxp3 expression in pancreatic carcinoma cells as a novel mechanism of immune evasion in cancer. Cancer Res.

[5] Merlo A, Casalini P, Carcangiu ML, Malventano C, Triulzi T, Mènard S, Tagliabue E, Balsari A (2009). FOXP3 expression and overall survival in breast cancer. J Clin Oncol.

[6] Zuo T, Wang L, Morrison C, Chang X, Zhang H, Li W, Liu Y, Wang Y, Liu X, Chan MW (2007). FOXP3 is an X-linked breast cancer suppressor gene and an important repressor of the HER-2/ErbB2 oncogene. Cell.

[7] Ladoire S, Arnould L, Mignot G, Coudert B, Rébé C, Chalmin F, Vincent J, Bruchard M, Chauffert B, Martin F (2011). Presence of Foxp3 expression in tumor cells predicts better survival in HER2-overexpressing breast cancer patients treated with neoadjuvant chemotherapy. Breast Cancer Res Treat.

[8] Wang L, Liu R, Li W, Chen C, Katoh H, Chen GY, McNally B, Lin L, Zhou P, Zuo T (2009). Somatic single hits inactivate the X-linked tumor suppressor FOXP3 in the prostate. Cancer Cell.

[9] Wu Y, Borde M, Heissmeyer V, Feuerer M, Lapan AD, Stroud JC, Bates DL, Guo L, Han A, Ziegler SF (2006). FOXP3 controls regulatory T cell function through cooperation with NFAT. Cell.

[10] Bettelli E, Dastrange M, Oukka M (2005). Foxp3 interacts with nuclear factor of activated T cells and NF-kappa B to repress cytokine gene expression and effector functions of T helper cells. Proc Natl Acad Sci USA.

[11] Chaudhry A, Rudra D, Treuting P, Samstein RM, Liang Y, Kas A, Rudensky AY (2009). CD4^+^ regulatory T cells control TH17 responses in a Stat3-dependent manner. Science.

[12] Ono M, Yaguchi H, Ohkura N, Kitabayashi I, Nagamura Y, Nomura T, Miyachi Y, Tsukada T, Sakaguchi S (2007). Foxp3 controls regulatory T-cell function by interacting with AML1/Runx1. Nature.

[13] Chen C, Rowell EA, Thomas RM, Hancock WW, Wells AD (2006). Transcriptional regulation by Foxp3 is associated with direct promoter occupancy and modulation of histone acetylation. J Biol Chem.

[14] Li B, Samanta A, Song X, Iacono KT, Bembas K, Tao R, Basu S, Riley JL, Hancock WW, Shen Y (2007). FOXP3 interactions with histone acetyltransferase and class II histone deacetylases are required for repression. Proc Natl Acad Sci USA.

[15] Zheng Y, Josefowicz SZ, Kas A, Chu TT, Gavin MA, Rudensky AY (2007). Genome-wide analysis of Foxp3 target genes in developing and mature regulatory T cells. Nature.

[16] Rudra D, deRoos P, Chaudhry A, Niec RE, Arvey A, Samstein RM, Leslie C, Shaffer SA, Goodlett DR, Rudensky AY (2012). Transcription factor Foxp3 and its protein partners form a complex regulatory network. Nat Immunol.

[17] Liang YJ, Liu HC, Su YX, Zhang TH, Chu M, Liang LZ, Liao GQ (2011). Foxp3 expressed by tongue squamous cell carcinoma cells correlates with clinicopathologic features and overall survival in tongue squamous cell carcinoma patients. Oral Oncol.

[18] Sadlon TJ, Wilkinson BG, Pederson S, Brown CY, Bresatz S, Gargett T, Melville EL, Peng K, D’Andrea RJ, Glonek GG (2010). Genome-wide identification of human FOXP3 target genes in natural regulatory T cells. J Immunol.

[19] Wang X, Seed B (2003). A PCR primer bank for quantitative gene expression analysis. Nucleic Acids Res.

[20] Hancock WW, Ozkaynak E (2009). Three distinct domains contribute to nuclear transport of murine Foxp3. PLoS One.

[21] Medzhitov R (2010). Inflammation 2010: New adventures of an old flame. Cell.

[22] Cantini G, Pisati F, Mastropietro A, Frattini V, Iwakura Y, Finocchiaro G, Pellegatta S (2011). A critical role for regulatory T cells in driving cytokine profiles of Th17 cells and their modulation of glioma microenvironment. Cancer Immunol Immunother.

